# The CombinADO study to assess the impact of a combination intervention strategy on viral suppression, antiretroviral therapy adherence, and retention in HIV care among adolescents and young people living with HIV: protocol for a cluster-randomized controlled trial

**DOI:** 10.1186/s13063-021-05943-w

**Published:** 2021-12-27

**Authors:** Phepo Mogoba, Maia Lesosky, Allison Zerbe, Joana Falcao, Claude Ann Mellins, Christopher Desmond, Carlos Arnaldo, Bill Kapogiannis, Landon Myer, Elaine J. Abrams

**Affiliations:** 1 Division of Epidemiology & Biostatistics, School of Public Health & Family Medicine, University of Cape Town, Level 5, Falmouth Building, Anzio Road, Cape Town, South Africa; 2 ICAP at Columbia University, Mailman School of Public Health, New York, USA; 3 HIV Center for Clinical and Behavioral Studies, Department of Psychiatry, New York State Psychiatric Institute and Columbia University Irving Medical Center, New York, NY USA; 4 Centre for Rural Health, University of KwaZulu Natal, Durban, South Africa; 5 Centro de Estudos Africanos, Universidade Eduardo Mondlane, Maputo, Mozambique; 6 Eunice Kennedy Shriver National Institute of Child Health and Human Development, Bethesda, MD USA; 7 Department of Pediatrics, Vagelos College of Physicians and Surgeons, Columbia University, New York, USA

**Keywords:** Adolescent, Youth, HIV, Mozambique, Cluster-randomized controlled trial, Multi-component intervention, Viral suppression, ART adherence, Retention, Implementation

## Abstract

**Background:**

Adolescents and youth living with HIV (AYAHIV) have worse HIV outcomes than other age groups, particularly in sub-Saharan Africa (SSA). AYAHIV in SSA face formidable health system, interpersonal- and individual-level barriers to retention in HIV care, uptake of ART, and achievement of viral suppression (VS), underscoring an urgent need for multi-component interventions to address these challenges. This cluster-randomized control trial (cRCT) aims to evaluate the effectiveness and monitor implementation of a community-informed multi-component intervention (“CombinADO strategy”) addressing individual-, facility-, and community-level factors to improve health outcomes for AYAHIV.

**Methods:**

This trial will be conducted in 12 clinics in Nampula Province, Northern Mozambique. All clinics will implement an optimized standard of care (control) including (1) billboards/posters and radio shows, (2) healthcare worker (HCW) training, (3) one-stop adolescent and youth-friendly services, (4) information/motivation walls, (5) pill containers, and (6) tools to be used by HCW during clinical visits. The CombinADO strategy (intervention) will be superadded to control conditions at 6 randomly selected clinics. It will include five additional components: (1) peer support, (2) informational/motivational video, (3) support groups for AYAHIV caregivers, (4) AYAHIV support groups, and (5) mental health screening and linkage to adolescent-focused mental health support. The study conditions will be in place for 12 months; all AYAHIV (ages 10–24 years, on ART) seeking care in the participating sites will be exposed to either the control or intervention condition based on the clinic they attend. The primary outcome is VS (viral load < 50 copies/mL) at 12 months among AYAHIV attending participating clinics. Secondary outcomes include ART adherence (self-reported and TDF levels) and retention in care (engagement in the preceding 90 days). Uptake, feasibility, acceptability, and fidelity of the CombinADO strategy during implementation will be measured. Trial outcomes will be assessed in AYAHIV, caregivers, healthcare workers, and key informants. Statistical analyses will be conducted and reported in line with CONSORT guidelines for cRCTs.

**Discussion:**

The CombinADO study will provide evidence on effectiveness and inform implementation of a novel community-informed multi-component intervention to improve retention, adherence, and VS among AYAHIV. If found effective, results will strengthen the rationale for scale up in SSA.

**Trial registration:**

ClinicalTrials.gov NCT04930367. Registered on 18 June 2021

**Supplementary Information:**

The online version contains supplementary material available at 10.1186/s13063-021-05943-w.

## Background

HIV burden among adolescents and young adults (aged 10–24) worldwide is substantial, with an estimated 1.7 million adolescents aged 10–19 years living with HIV in 2019 and 460,000 adolescents and youth newly infected in the same year [1]. An estimated 85% of all adolescents and young people living with HIV (AYAHIV) reside in sub-Sharan Africa [SSA] [2]. This is concerning given that SSA has a young population, with 30–35% of the total population between 10 and 24 years of age [1]. While increased availability and access to antiretroviral therapy (ART) have significantly reduced pediatric mortality, healthcare workers (HCWs) face challenges in meeting the complex needs of the large number of surviving AYAHIV who must learn to manage HIV, a chronic, highly stigmatized, and transmittable illness. Furthermore, adolescents and young adults newly acquiring HIV in these developmental stages experience overlapping but distinct and equally complex needs that require tailored services.

AYAHIV in SSA face formidable health system, interpersonal- and individual-level barriers to retention in HIV care, uptake of ART, and achievement of viral suppression (VS) and worse outcomes compared to other age groups within HIV programs have been identified [3]. Results from the Population HIV Impact Assessments (PHIA) in four SSA countries, Zambia, Zimbabwe, Malawi, and Eswatini, demonstrated lower rates of HIV testing, ART uptake, and VS among 15–19-year-olds compared with adults [4]. Previous studies conducted in SSA have identified multiple factors impacting treatment outcomes in AYAHIV, many in common with adult populations but compounded by the challenges of adolescence and early adulthood, including psychosocial factors and lack of social support, as well as factors relating to health systems such as mistrust of HCWs and concerns about stigma and discrimination, especially when accessing sexual and reproductive health (SRH) services [5–11]. Evidence from our formative survey among 213 AYAHIV ages 10–19 years surveyed in Nampula Province Mozambique showed a high proportion of participants with missed ART doses and high levels of viremia, and over two-thirds self-reported challenges with ART adherence [12–14].

There are few evidence-based interventions designed to promote increased retention in care, ART adherence, and VS among AYAHIV in low- to middle-income countries (LMIC). Peer support and support groups are the most widely used approaches, seen in Malawi with adolescent-centered differentiated service delivery (DSD), which integrates structured adolescent-focused groups, adherence counseling, and social activities into clinic visits which are aligned with school hours which has been associated with higher retention; and a peer-supported community-based DSD model in Zimbabwe that demonstrated significantly improved VS among AYAHIV [15, 16]. In addition, counseling and education (individual, family, group multidisciplinary with AYAHIV and/or caregivers) have been noted as modestly associated with improved adherence among AYAHIV [17].

Mozambique has the second-highest number of new HIV infections in Eastern and Southern Africa, high HIV burden among adolescents, and poor treatment coverage with progress towards the UNAIDS 95-95-95 targets for HIV epidemic control lagging behind other countries in the region [18]. HIV prevalence in Mozambique is 13.2%, with an estimated 1.8 million people living with HIV (PLHIV). More than 30% of the general population is aged between 10 and 24 years, and they face high rates of poverty and unemployment and poor access to education, all of which contribute to poor health outcomes [19]. Nationally, there are an estimated 143,000 AYAHIV, 10–19 years, only half of whom (54%) are currently on ART [20)]. Despite improving access to care and better outcomes for those initiated on ART, rates of VS remain low, especially among young men. Among AYAHIV ages 10–14, 15–19, and 20–24, VS among females is 64%, 72%, and 80%, respectively, and VS among males is 60%, 62%, and 75%, respectively [20–22]. Mozambique has a national package of adolescent and youth-friendly services (AYFS), including HIV prevention, care, and treatment, screening, and treatment for sexually transmitted infections (STIs), family planning, and intimate partner violence (IPV) services. However, in parts of Mozambique, AYFS have only been implemented in only a few health facilities [23]. These gaps underscore the need for developing a youth-focused multi-component combination intervention for AYAHIV in Mozambique.

Recognizing the paucity of evidence to inform the successful scale-up of quality services for children and adolescents living with HIV, WHO and CIPHER developed a global research agenda that emphasized the need for effective strategies to improve adherence, retention, SRH outcomes, and service delivery models to improve outcomes along the HIV continuum, including peer interventions and DSD models [24]. Furthermore, the National Institute of Child Health and Development (NICHD) requested research proposals to generate much needed scientific innovation to yield effective public health interventions for young people affected by HIV in resource-limited settings, the Prevention and Treatment through a Comprehensive Care Continuum for HIV-affected Adolescents in Resource-Constrained Settings (PATC^3^H) program. In collaboration with the Mozambique Ministry of Health (MISAU), researchers from ICAP at Columbia University were awarded a grant to develop and evaluate a complex, multi-component intervention to improve HIV related health outcomes of AYAHIV in Mozambique (CombinADO).

The CombinADO project is unique in its focus on shifting the paradigm of AYAHIV services from one of fear and secrecy to one of hope and inclusion. The formative work conducted during the initial phase of CombinADO has enhanced the evidence base with rich information regarding the needs of AYAHIV and best practices around AYAHIV engagement to help increase the adoption of interventions. Building on this, collaborations with a broad array of local stakeholders have led to the design and development of a multi-component intervention strategy that will be trialed in this study.

### Objectives

The primary objective is to evaluate the efficacy of the intervention (hereafter “*CombinADO strategy*”) on the rate of HIV VS among AYAHIV receiving HIV care at 12 health facilities in Nampula, Mozambique. The secondary objectives are (1) to examine the efficacy of the CombinADO strategy on the rate of retention in care among AYAHIV receiving HIV care at 12 health facilities in Nampula Mozambique; (2) to evaluate the efficacy of the CombinADO strategy on the rate of adherence to ART among AYAHIV receiving HIV care at 12 health facilities in Nampula Mozambique; (3) to assess the uptake, feasibility, and acceptability of the CombinADO strategy at 12 health facilities in Nampula Mozambique; and (4) to estimate the cost and incremental cost-effectiveness of the CombinADO strategy at 12 health facilities in Nampula, Mozambique.

## Methods and analysis

The study is conducted as part of the NICHD funded PATC^3^H consortium. The goal of PATC^3^H is to generate the needed scientific innovation that will yield effective public health interventions for adolescents (10–24 years) affected by HIV in Brazil and SSA [25]. This protocol was written following the Standard Protocol Items Recommendations for Interventional Trials (SPIRIT) guidelines (see Additional file 1: SPIRIT checklist).

### Study design

This is a cluster-randomized controlled trial (cRCT) designed to compare the effectiveness of the CombinADO strategy versus optimized standard of care (SOC) on VS, ART adherence, and retention in HIV care among AYAHIV ages 10–24 years attending participating health facilities. Clinics are the units of intervention allocation and randomization; however, trial outcomes will be assessed at the individual level among AYAHIV. An independent statistician will generate the clinic randomization schema. All AYAHIV seeking care will be exposed to either the control condition or intervention condition based on the facility attended. Health facility staff will implement the various intervention components at both the intervention and control sites as the standard of care for AYAHIV.

### Study setting

The study will be conducted in 12 study sites across Nampula Province, Northern Mozambique. The HIV prevalence among young people 15–24 years in this province is estimated at 4.1%, with only 16% of Nampula clinics currently offering youth-friendly services [23]. Clinics were eligible to be selected for participation based on location (within a 4-h drive from Nampula city), patient volume (at least 220 AYAHIV ages 10–24 currently active on ART), and one-stop-shop adolescent-friendly service model care. Out of 15 eligible clinics, 12 of these were randomly selected for participation by an independent biostatistician.

### Participants and recruitment

Eligible study participants include AYAHIV, AYAHIV caregivers, HCW, and key informants (KI). The eligibility criteria for the different groups are outlined in Table 1. The schedule for the various study activities for study participants is shown in Table 2.

**Table 1:** Inclusion and exclusion criteria and the anticipated sample size of all the CombinADO study participants

Participant	Inclusion	Exclusion	Anticipated sample size
AYAHIV	HIV-positive, per medical records and confirmed by health facility staff Age 10–24 years Registered as a patient at the study site Aware of HIV+ status Provision of signed and dated informed consent form for adolescents aged 18 and above For adolescents aged 12–17, informed assent and parental informed consent to participate in the study For adolescents aged 10–11, parental consent to participate in the study Stated willingness to comply with all study procedures	Has an acute medical condition requiring immediate medical care	*N* = a minimum of 1200 and a maximum of 2400 for the post-intervention assessment *N* = a minimum of 360 and a maximum of 720 exit interviews
Caregivers	Age 18 years and older Attending a caregiver-specific support group Willingness to be audio-recorded	None	*N* = a minimum of 90 and a maximum of 120 semi-structured interviews
HCW	Age 18 years and older Involved in the provision, management, or oversight of adolescent-focused HIV services at the 12 specified study sites Willingness to be audio-recorded	None	*N* = a minimum of 70 and a maximum of up to 112 semi-structured interviews *N* = a minimum of 35 and a maximum of up to 50 costing questionnaires
KI	Age 18 years and older Involved in the provision, management, or oversight of adolescent-focused HIV services at the 12 specified study sites Willingness to be audio-recorded	None	*N* = a minimum of 32 and a maximum of 60 semi-structured interviews

**Table 2:** Study assessment schedule for all the CombinADO participants

	Pre-intervention	Intervention implementation	Post-intervention
**Adolescents and young adults living with HIV**			
Eligibility assessment		X	X
Informed consent/assent		X	X
Exit interviews		X	
Survey questionnaire			X
Specimen collection^a^			X
**Adolescent caregivers**			
Eligibility assessment		X	
Informed consent		X	
Semi-structured interviews		X	
**Healthcare workers and key informants**			
Eligibility assessment^b^	X		
Informed consent ^b^	X		
Semi-structured interviews	X	X	X
Costing questionnaires	X	X	
**Site-level activities**			
Direct clinic observation		X	
Collection of process indicators (monitoring of intervention fidelity/uptake)		X	
Abstraction of routine data			X

^a^Real-time viral load testing and stored samples

^b^In the event that a HCW or KI leaves a study site, the new staff member at the site will be enrolled as a replacement

#### AYAHIV

Up to 2400 AYAHIV (maximum of 200 AYAHIV per site) will be recruited to take part in a one-time post-intervention assessment at 12 months post-intervention implementation and up to 720 AYAHIV (maximum of 60 per site) in exit interviews during intervention implementation. Clinic staff will work with on-site study staff to review the daily schedule and list potentially eligible AYAHIV who have appointments. During the clinic visits at the 12 sites, clinic staff will consecutively approach and directly present the study to those 18 years and older or the adult caregiver for adolescents younger than 18. Both interested AYAHIV and caregivers will be referred to the study staff for recruitment. The study staff will give AYAHIV more details on the study procedures, screen AYAHIV for eligibility using criteria in Table 1, and finally obtain informed consent from AYAHIV 18 years and older or assent from adolescents aged 12–17 of consenting caregivers. Eligible adolescents aged 10–11 will be given information sheets that describe the study, and consent for their participation will be obtained from their caregivers. The study staff will note reasons for ineligibility and/or refusal and AYAHIV will be replaced until target sample is reached at each site.

#### Caregivers

Up to 120 AYAHIV caregivers (up to 20 per intervention site) will be recruited to participate in semi-structured interviews after attending caregiver-specific support groups during the intervention implementation. The facilitators leading the support groups will identify caregivers to participate in post-support group interviews, present the potential participation in the interviews directly to caregivers, and refer interested caregivers to on-site study staff to learn more about the interview procedures. If interested in participating, caregivers will be screened based on the criteria in Table 1, and those found eligible and agree to participate will undertake the informed consent process.

#### HCW and KI

Up to 112 HCWs and 60 KIs will be recruited to participate in semi-structured interviews before, during, and after intervention implementation. In addition, a subset of up to 50 HCWs enrolled in semi-structured interviews will be asked to complete an additional costing questionnaire at multiple time points before and during intervention implementation. The study team will meet with the administration at each study site to gain permission to invite HCWs to participate in interviews. Once approval is granted, the study will be introduced to the HCWs at routine staff meetings. Staff interested in the study will receive additional information about the interview from the study team and, if interested in participating, HCWs will be screened based on the criteria in Table 1. Those who are deemed eligible and agree to participate will undertake the informed consent process. HCWs will be assured that their decision to participate will not impact their employment status.

The study team will compile a list of KIs who can be invited to participate in pre/post-intervention interviews. KIs will include ICAP project staff, health facility directors, and staff from regional health offices and MISAU, who will provide their perceptions about implementing the study intervention at a managerial level. Once contacted, all KIs who express interest in learning more will be given basic interview participation information. If interested, they will be screened based on the criteria in Table 1. Those who are eligible will undertake the informed consent process.

For all other participants (HCWs, KIs, caregivers), purposeful sampling will be used, recruiting participants that have been directly involved in the implementation and who are interested in sharing their views with our study interviewers.

### Interventions

The intervention design is based on extensive formative research with AYAHIV, HCWs, AYAHIV caregivers, and other key stakeholders in this setting. Preliminary work included implementation science (quantitative surveys, qualitative interviews) and human-centered design methods, under which AYAHIV and stakeholders including AYAHIV participated in an intensive, iterative process of testing and refinement to develop a multi-component intervention strategy, the CombinADO strategy [14]. Once developed, this strategy was piloted with AYAHIV, caregivers, and HCWs to demonstrate the various intervention components’ acceptability and feasibility. Based on this formative phase’s findings, we designed a multi-component package of intervention modalities that will be delivered as part of enhanced services for AYAHIV at intervention sites. Both the control and intervention conditions will be in place at the study sites for 12 months. A complete list of control and intervention components and each component’s rationale is outlined in Table 3.

**Table 3:** CombinADO study arm components during the 12 months of implementation

Component	Rationale	Study arm	
		Control	Intervention
Radio ads	Engaging radio mini shows that address community stigma and medical literacy through busting common myths with humor and building empathy with heartfelt storytelling	X	X
Community sensitization campaign	Large-scale, infographic billboards and posters located in public areas and secondary schools to address stigma and medical literacy and promote community support for AYAHIV	X	X
Informational posters	Large-scale, infographic posters located in clinic waiting areas to normalize HIV and build confidence in treatment	X	X
Motivation walls	Interactive, patient-generated posters located in the consultation room where patients can post words and phrases about themselves and their futures	X	X
Pill boxes	A discreet pill container to support ART adherence	X	X
CombinADO-specific AYAHIV training	Comprehensive in-service training for healthcare workers	X	X
One-stop shop	Combined adolescent and HIV services	X	X
Treatment toolkit	A guide to clinic visits and discussions on Art and viral load monitoring to help HCW better communicate with patients	X	X
Self-reflection kit	A simple handout for providers to help patients reflect on their ART progress and understand the concept of viral load as a measure of ART success	X	X
Peer support at clinical level	Peer exposure to examples of AYAHIV openly living with HIV and opportunities to share their experiences with HIV in one-on-one interactions with other AYAHIV during clinic visits		X
Informational and motivational video	An informational and motivational video that in simple language with engaging graphics that (a) demystifies and simplifies HIV, ART, and viral load and (b) emphasizes that people can live long, healthy lives		X
Support groups for caregivers of AYAHIV	A learning, support, and empowerment group for caregivers of AYAHIV. Through monthly gatherings, the program aims to foster confidence and equip caregivers with strategies to support AYHIV adherence journey		X
Support groups for AYAHIV	A peer-to-peer learning, support, and empowerment group to address loss of hope and improve medical literacy. Through biweekly gatherings, the program aims to foster belonging and confidence, equipping young people and caregivers with strategies to navigate the adherence journey		X
Mental health screening and linkage to adolescent-focused mental health support	HCWs will be trained in the use of a brief mental health screening tool focusing on depression, anxiety, and post-traumatic stress disorder. Mental health service providers at each facility will be trained and supported to provide diagnostic and mental health support to youth with positive screens who agree to further evaluation		X

#### Description of the control condition (optimized SOC)

**Fig. 1:**
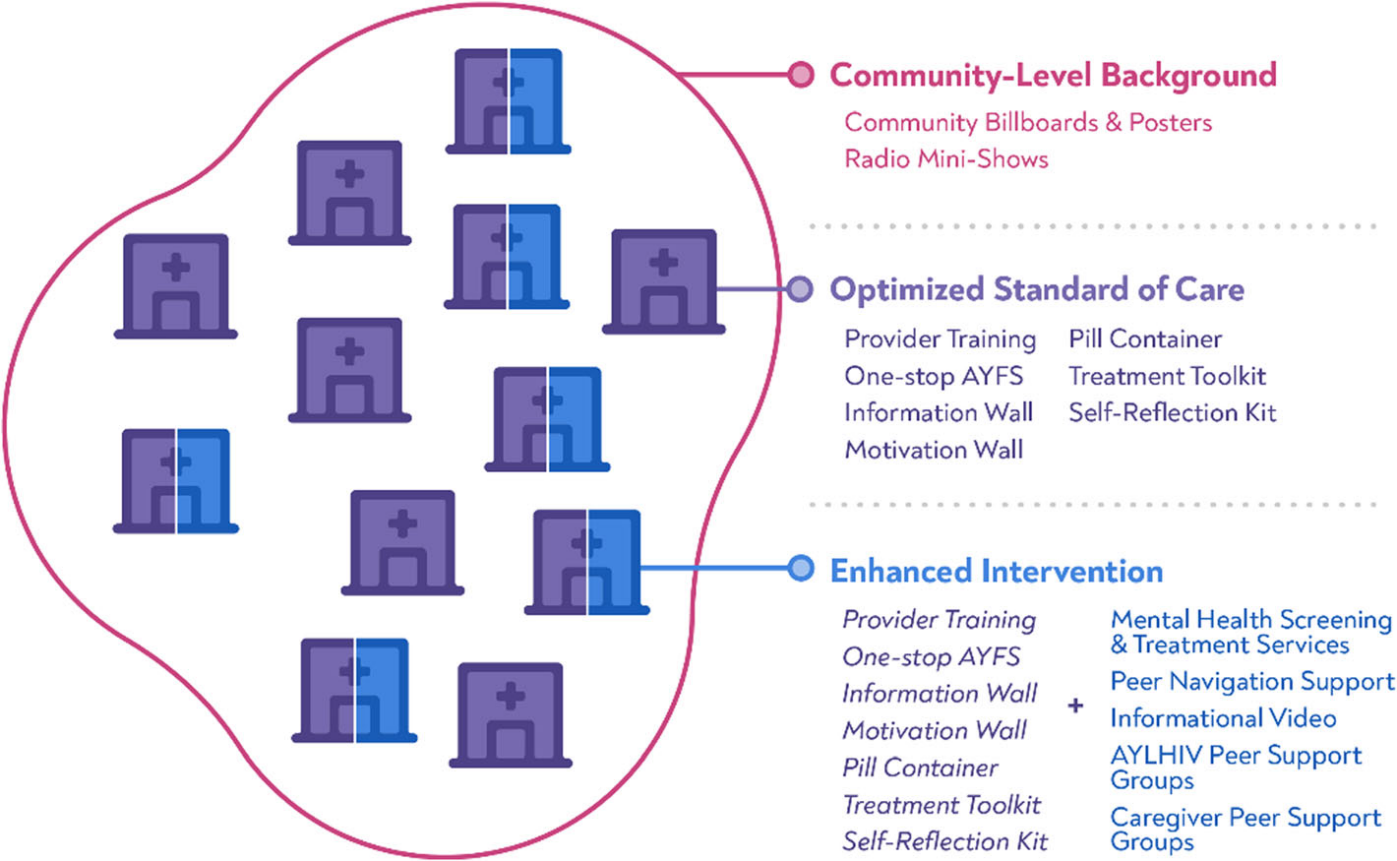
CombinADO study design for the clinic-level clusters: purple areas indicate the control conditions (optimized standard of care) and the blue indicates intervention conditions (enhanced intervention)

The control condition will be implemented at all 12 clinic sites and includes a set of interventions aimed at optimizing the national standard of care, including (1) billboards/posters and radio shows, (2) healthcare worker training, (3) one-stop adolescent and youth-friendly services, (4) information/motivation walls, (5) pill containers, and (6) tools to be used by clinic staff during clinical visits (Fig. 1 and Table 3).

#### Description of the intervention condition (enhanced intervention)

The intervention condition will be superadded to the control condition at six randomly selected clinic sites and includes the following five additional intervention components: (1) peer support, (2) informational and motivational video, (3) support groups for caregivers of AYAHIV, (4) support groups for AYAHIV, and (5) mental health screening and linkage to adolescent-focused mental health support (Fig. 1 and Table 3).

### Randomization and blinding

Randomization will be balanced 1:1, with an equal number of study sites randomized to each arm.

A randomization list will be prepared by the independent statistician and passed to the study team. The intervention allocation is open to the study team and will not be blinded to investigators or HCWs and AYAHIV participating in the trial. However, all staff involved in data analysis will be blinded to arm allocation, including the trial statistician. To facilitate this, we will maintain separate databases for variables collected only at intervention sites (including process evaluation measures), independent from data on primary and secondary outcomes collected at intervention and control sites in an identical manner. In addition, laboratory staff involved in measuring the primary outcome will also be blinded to arm allocation.

The independent statistician will maintain randomization codes, and unblinding of the analysis team will occur only after final results using dummy randomization allocation have been completed by the study statistician. As the study team is unblinded to allocation, they will be able to address any issues related to adverse events. If the trial statistician or other personnel working in data analysis become unblinded prematurely for any reason, the principal investigator (PI) and investigator team will be notified immediately. Potential biases introduced through inadvertent unblinding will be examined, along with strategies to mitigate the impact of this on trial reporting.

### Study outcomes

The trial outcomes are described in Table 4 and include effectiveness outcomes and implementation outcomes summarized below.

**Table 4:** Study objectives and justification for the study endpoints

Objectives	Endpoints	Justification for endpoints	Putative mechanisms of action
**Primary**			
To evaluate the efficacy of the CombinADO strategy on the rate of HIV viral suppression among AYAHIV receiving HIV care at 12 health facilities in Nampula Mozambique	Viral suppression < 50 copies/mL at 12 months among AYAHIV in care at intervention and control sites	HIV viral suppression is a major goal of effective ART services, is readily measured, and is a well-established as the gold standard outcome to evaluate HIV treatment interventions	The CombinADO strategy is hypothesized to increase viral suppression through increases in retention in care and/or adherence to ART in the target population
**Secondary**			
To evaluate the efficacy of the CombinADO strategy on the rate of retention in care among AYAHIV receiving HIV care at 12 health facilities in Nampula Mozambique	Retention in care defined as objective evidence of engagement in the preceding 90 days among AYAHIV in care at intervention and control sites	Increases in retention in care is a causal intermediate (mediator) through which the CombinADO strategy may increase HIV viral suppression	Different elements of the CombinADO strategy are likely to support AYAHIV’s retention in care during the intervention period
To evaluate the efficacy of the CombinADO strategy on the rate of adherence to ART among AYAHIV receiving HIV care at 12 health facilities in Nampula Mozambique	Self-reported using the Wilson measure; TDF levels on a subset of participants in care, selected retrospectively in a case-control substudy comparing AYAHIV who have VL < 50 copies/mL, versus those with VL ≥ 50 copies/mL	Increases in ART adherence is a causal intermediate (mediator) through which the CombinADO strategy may increase HIV viral suppression	Different elements of the CombinADO strategy are likely to support AYAHIV’s adherence to ART during the intervention period
To assess the uptake, feasibility, and acceptability of the CombinADO strategy at 12 health facilities in Nampula Mozambique	Uptake and utilization of intervention components by AYAHIV among AYAHIV in care at intervention and control sites; implementation fidelity by health care workers and key informants	Uptake and implementation of the intervention is an intermediate (mediator) through which the CombinADO strategy may increase HIV viral suppression	Inadequate uptake and implementation of the intervention and/or components can limit efficacy of the intervention
To estimate the cost and incremental cost-effectiveness of the CombinADO strategy at 12 health facilities in Nampula Mozambique	Incremental cost per additional case of viral suppression < 50 copies/mL at 12 months	HIV viral suppression provides an objective summary measure of intervention effectiveness	The CombinADO strategy is hypothesized to increase viral suppression through increases in retention in care and/or adherence to ART in the target population

#### Effectiveness outcomes

The primary outcome is viral suppression < 50 copies/mL at 12 months post-intervention implementation among AYAHIV at intervention and control sites, which will be assessed through real-time viral load testing.

Secondary outcomes include:

(1) ART adherence will be measured by using a validated Wilson self-report ART adherence measure administered on all AYAHIV participating in the one-time post-implementation assessment at 12 months post-intervention implementation [26, 27]. In addition, TDF levels will be measured on a subset of participants in care selected retrospectively in a case-control sub-study comparing AYAHIV who have VL < 50 copies/mL, versus those with VL ≥ 50 copies/mL. The Wilson measure has previously been implemented in this setting to assess ART adherence among AYAHIV during the first phase of the CombinADO study [12]. (2) Retention in care will be measured as objective evidence of engagement in the preceding 90 days among AYAHIV in care at intervention and control sites abstracted from routine clinic records.

#### Implementation outcomes

We will also monitor the implementation process’s uptake, feasibility, acceptability, and fidelity of the CombinADO strategy. This will measure uptake and utilization of intervention components by AYAHIV among AYAHIV in care at intervention and control sites and implementation fidelity by HCWs and KIs. Both acceptability and feasibility will be assessed qualitatively in interviews; fidelity will be assessed through monthly observational assessments and review of health facility registers conducted by study staff. Additional questionnaires related to cost/cost-effectiveness will also be conducted with a subset of HCWs at each site.

### Data collection

Trial outcomes comprise of data collected from (1) a questionnaire developed during the formative phase administered to AYAHIV in a private space by trained fieldworkers at the post-intervention assessment, (2) exit interviews with AYAHIV attending care at adolescent-focused services during intervention implementation, (3) semi-structured interviews with AYAHIV caregivers attending caregiver support groups, (4) semi-structured interviews with HCWs and KIs, (5) costing questionnaire with a subset of HCWs, (6) retrospective abstraction of AYAHIV medical records in the 12 participating facilities for data on HIV care and treatment, (7) collection of aggregate facility-level routinely collected data for retention, (8) observational data collected by study staff on time taken for clinic activities, (9) collection of process indicators on implementation intervention, and (10) blood sampling in AYAHIV at the post-intervention assessment for real-time viral load testing and dried blood spot (DBS) for future TDF-DP drug level testing.

All procedures for data collection (regardless of source) will be outlined in standard operating procedures (e.g., for DBS, specimen transport or questionnaire administration), from which all study staff will be trained ahead of study implementation at each site. With oversight from the Mozambique-based Study Coordinator, the CombinADO Field Manager will work with a dedicated team to ensure consistent administration (fidelity of delivery) of the study intervention across the 12 study sites during the 12 months of implementation.

The study team, including all staff collecting data from participants as well as participating HCWs, will be trained in an emergency protocol to be able to identify participants requiring additional support and follow-up (medical, social, psychological) as well as the process for referrals for existing support services that are in place at each health facility. A further description of measurements that will be collected through the different processes is outlined in Table 5.

**Table 5:** Study data collection sources, information collected, and timing of collection for all CombinADO study participants

Measurement	Information collected	Participants	Time
Survey questionnaire	o Basic demographic information, education, and vocation information o HIV, STI, ART, reproductive health beliefs and literacy o Adherence and adherence self-efficacy o Youth readiness for independent health care o Relationship/marital status o Mental health o Life events o Substance use o Sexual risk o Reproductive health, including pregnancy and parenthood o Interpersonal violence o Partner, family, and social support o HIV stigma/disclosure o Experience with COVID-19 o Uptake of CombinADO strategy intervention components o Feasibility, acceptability, and appropriateness of all strategy intervention components	AYAHIV	T3
Exit interviews	o Experiences and satisfaction with the services received and various intervention components	AYAHIV	T2
Semi-structured interviews	o Perceived benefits and challenges of attending the support groups o Comfort taking part in the support group o Satisfaction with current support groups structure o Recommendations for refining the existing support groups	Caregivers	T2
	o Experience implementing the intervention, including time spent providing care to AYAHIV o Impact of the intervention on facility and delivery of health services o Met and unmet needs of the AYAHIV population seeking services o Adoption, implementation, maintenance, reach, feasibility, acceptability appropriateness, and fidelity of the intervention various components	HCWs and KIs	T1–T3
Costing questionnaire	o The daily schedule at the health facility o Time spent working across various clinic activities, including consultations with AYAHIV	HCWs	T1 and T2
Blood sampling	o Blood for real-time viral testing o Dried blood spot (DBS) samples for TDF-DP drug-level testing	AYAHIV	T3
Abstraction of AYAHIV routine data	o Date of HIV diagnosis o ART start date o Current ART regimen o Intercurrent hospitalizations o Most recent HIV RNA viral load and CD4 results o Pharmacy dispensing and refill information o Most recent visit date o Retention in care	Clinic records	T3
Monitoring of implementation process	o Number and duration of support groups conducted o Number of AYAHIV and caregivers attending support groups (new/returning) o Curriculum materials and intervention content delivered o Number of AYAHIV expected within the clinic and number interacting with various components of the intervention package o Number of clinic-based peers trained/retained o Number of support group facilitators trained/retained o Number of HCWs trained on new service components o Number of radio and print materials posted/broadcast in the community	Site staff	T2

Time: *T1* pre-intervention, *T2* intervention implementation, *T3* post-intervention implementation

### Data management

During the study, study staff will have access to research records. In addition, members of the Mozambique Ethics and Scientific Review Committee (CNBS), the Columbia University Irving Medical Center (CUIMC) IRB, study monitors and other additional local and US regulatory agencies like the Office for Human Research Protections (OHRP) as well as the study sponsor, National Institutes of Health (NIH), may look at the research records.

All digital data collection tools will be encrypted, password protected, and collected from the staff at the end of each day to be stored in locked filing cabinets at the study sites. All datasets will be stored on SurveyCTO during data collection and accessed on password-protected computers certified by Columbia University IT. All hard copies of all source documents, data collection tools, and study logs be kept in a secure locked cabinet in a locked office at the study site. No participant identifying information will be documented on any of these data sources; participants will only be identified by a unique participant ID (PID). All documents with personal identifying information will be stored separately from all study data and accessible to specific research staff (data collection staff, study coordinator, and study monitors). All DBS samples collected at the post-intervention assessments will be stored for the duration of this study at the INS lab based in Nampula until required for TDF-DP drug level testing planned for end of the study . No blood samples will be stored or shared for future use outside of this protocol.

At the end of the study, all records will continue to be kept in a secure location for as long a period as dictated by the reviewing IRB, Institutional policies, or sponsor requirements.

### Data analysis

#### Populations for analyses

The primary outcome (and secondary adherence outcome) will use an intention to treat population of all participants consenting to the post-intervention assessment who complete a questionnaire and VL test. The per-protocol population for the primary outcome (and secondary adherence outcome) will define exposure to the intervention based on AYAHIV-stated engagement in one or more component of the CombinADO intervention during interviews at the post-intervention assessment. The secondary retention in care endpoint will use the eligible (based on inclusion criteria) population of all AYAHIV registered at the clinic site during a 15-month period extending from 6 months before the start of the intervention period to the end of the intervention period. Data for the secondary retention in care endpoint will use routine data abstracted from clinic records. This definition will exclude AYAHIV, who newly initiated ART at the site in the final 6 months before the end of the intervention period.

#### Statistical analysis

Descriptive statistics will be presented with frequency (percent) or median (interquartile range) depending on the type and will provide overall and by allocation arm. Review of data distributions will be undertaken prior to applying any inferential statistics requiring specific assumptions and methods revised or corrective procedures undertaken. Covariates and specific details of analysis will be fully specified before database closure in a detailed SAP developed by the study statistician and study team. All statistical tests will be two-sided with nominal *α* = 0.05. Study endpoints are binary and will be approached in a similar way. The primary outcome analysis will compare AYAHIV virologic suppression at 12 months post-intervention implementation in intervention versus control sites using generalized estimating equations under a binomial model, with a log link and robust standard errors, providing unbiased marginal estimates of the intervention effect while adjusting for within-cluster correlations. Clustering will be by site using an exchangeable correlation structure. Estimates will be reported as risk ratios (RR) with robust 95% confidence intervals and *p*-values assessed at *α* = 0.05 for all comparisons. No additional adjustment variables will be included in the primary analysis. In subsidiary analyses, we will include covariate adjustment based on confounders selected a priori using direct acyclic graphs. Sensitivity analysis will address potential heterogeneity in clustering by evaluating endpoint models under different assumed correlation structures. Missing outcome data will be handled by complete case analysis in the first case and then by sensitivity analysis substituting alternate outcome values for missing data. Missing data in covariates will be handled by multiple imputations using chained equations. Analysis of secondary endpoints is not conditional on the outcome of the primary endpoint. Secondary endpoints will be approached identically as the primary endpoint. Planned subgroup analyses, which include by age group [10–14, 15–19, 20–24], sex, pregnancy status during the intervention period among females, duration of prior ART use, and ART regimen, will be carried out for primary and secondary outcomes using the same approach as the previously described. Exploratory analyses will include:

- For analyses of primary and secondary endpoints, application of generalized linear mixed effects models instead of GEE in the case of heterogeneity between sites.
- Alternate definitions of endpoints:
  - Primary endpoint: viral suppression threshold at 1000 copies/mL
  - Secondary outcome (adherence): alternate thresholds to define adherent to ART based on preliminary analyses
  - Secondary outcome (retention): alternate thresholds to define retained on ART based on preliminary analyses

### Sample size and power

#### Primary outcome

The primary outcome analysis will compare AYAHIV virologic suppression at 12 months post-intervention, between intervention versus control sites using generalized estimating equations with robust standard errors, providing unbiased marginal estimates of the intervention effect while adjusting for within-cluster correlations.

For the primary outcome, sample size estimates are based on the following assumptions: 2-sided superiority test with 90% power at *α* = 0.05, clinic sizes of 220 AYAHIV in active care per clinic (range, 113–590), levels of viral suppression < 50 copies/mL of 65% in control clinics (current data estimates this proportion at 48–68%), *ICC* = 0.05 (range, 0.04–0.08). Thus, we estimate that 12 clusters sampling at least 100 AYAHIV each on average will provide > 80% power to detect an absolute increase of 16% in virologic suppression in the intervention versus control clinics.

#### Secondary outcomes

##### Adherence

Projected statistical power for comparing the secondary adherence outcomes between intervention versus control sites uses the same assumptions as above, thus having similar expectations around achieved power and minimal detectable differences.

##### Retention

Projected statistical power for the comparison between arms for the secondary retention outcomes between intervention versus control sites assumes clinic “in care” population sizes of an average of 220 AYAHIV in active care (12-month period) for a total analysis size = 2640 across 12 sites and an assumed proportion retained in care (90-day window) of 50–60% under control conditions.

### Ethical considerations and trial management

The trial was approved by the Columbia University of Irving Medical Center Institutional Review Board (CU-IRB) and the Comite Naticional De Bioetica Para A Saude (CNBS). Participant confidentiality and privacy are strictly held in trust by the participating investigators, staff, safety and oversight monitor(s), and study sponsor. This confidentiality is extended to the data being collected as part of this study. Data will be de-identified and stored on password-protected computers certified by Columbia University IT and only accessible to the study team.

Safety oversight will be under the direction of a Data and Safety Monitoring Board (DSMB) composed of individuals with the appropriate expertise, including HIV treatment, adolescent care, health systems evaluations, HIV service delivery, biostatistics, and/or trial design. Members of the DSMB will be independent of the study conduct and free of conflict of interest. The DSMB will meet at the beginning of the planned study and then at least once a year to review the study progress. The DSMB will operate under the rules of an approved charter that will be written and reviewed at the organizational meeting of the DSMB. The approved CombinADO Study Progress and Safety Monitoring Plan details the types of reports that the DSMB will assess throughout the study. In addition, the DSMB will provide its input to the National Institute of Child Health & Human Development (NICHD).

In addition, clinical site monitoring will be conducted to ensure that the rights and well-being of trial participants are protected; that the reported trial data are accurate, complete, and verifiable; and that the conduct of the trial is in compliance with the currently approved protocol/amendment(s), with International Council on Harmonisation Good Clinical Practice (ICH GCP), and with applicable regulatory requirement(s). Clinical site monitoring for this study will be performed by Westat in accordance with all of the procedures outlined in the Site Monitoring Plan for the PATC^3^H Program [25].

Any amendment to the protocol will require review and approval by the reviewing Institutional Review Board (IRB) before the changes are implemented to the study. Protocol deviations will be sent to the reviewing IRB per their policies. The site staff will be responsible for knowing and adhering to the reviewing IRB requirements.

### Dissemination plan

Data generated from the CombinADO project will be shared per the PATC^3^H Data and Recourse Sharing Policy, finalized in September 2019. Per this policy, CombinADO will utilize NICHD’s Data and Specimen Hub (DASH) as the repository for all data. Furthermore, this study will also comply with Clinical Trials Registration and Results Information Submission rule. As such, this trial has been registered at ClinicalTrials.gov, and the result information from this trial will be submitted to ClinicalTrials.gov. Also, every attempt will be made to publish results in peer-reviewed journals.

## Discussion

There is an urgent need for effective strategies to support treatment outcomes among AYAHIV, especially in low-resource settings. Most existing interventions are unidimensional and often insufficient to address the many complex challenges faced by AYAHIV. This trial assesses whether a community-informed multi-component intervention (the “CombinADO strategy”) addressing individual-, facility-, and community-level factors improves health outcomes for AYAHIV engaged in HIV services. The research will provide timely evidence of the design and effectiveness of a multi-component package of intervention modalities with the potential to improve critical outcomes, including retention, ART adherence, and VS among AYAHIV. Furthermore, information gained will inform the evidence-based and future implementation for this type of youth-friendly multi-component intervention strategy. We believe using a cRCT for this study will contribute to this study’s rigor, given the combination intervention evaluated as some components of the intervention will occur at the facility or site level. Individual randomization would lead to significant contamination, limiting the ability to measure any intervention effect. If found effective, results would strengthen the CombinADO strategy’s potential to be scaled up across Mozambique and similar settings. Also, this study benefits from the evidence base with rich information regarding the needs of AYAHIV and best practices around AYAHIV engagement gained from interactions with a broad array of stakeholders during the initial phase and is likely to increase adoption. We acknowledge a few limitations to this study. Firstly, contamination between sites will not be controlled for during implementation; however, the implementation process will be documented for potential barriers and facilitators to inform the analysis. Finally, we expect that the ongoing COVID-19 pandemic may contribute several operational challenges to implementing the trial; nevertheless, we believe this work will generate real-world lessons applicable to other resource-limited settings.

## Trial status

The trial presented here is aligned with IRB protocol version 1.3, approved on 02 August 2021. The recruitment started on 13 September 2021 and is anticipated to be completed in December 2022.

## Supplementary Information

**Additional file 1.** SPIRIT checklist

## Data Availability

Per study sponsor requirements, once the study is complete, all de-identified, archived data will be transmitted to and stored at NICHD’s Data and Specimen Hub (DASH) for use by other researchers, including those outside of the study: https://dash.nichd.nih.gov/. Permission to transmit data to DASH will be included in the informed consent/assent and information sheet. Data transmitted to NICHD’s DASH repository will only include that collected via surveys and interviews with study participants. Data collection tools are available upon reasonable request to the corresponding author or contacts listed in Clinicaltrials.gov.

## Abbreviations

ART: Antiretroviral therapy
AYAHIV: Adolescents and young people living with HIV
AYFS: Adolescent and youth friendly services
cRCT: Cluster-randomized control trial
DBS: Dried blood spot
DSD: Differentiated service delivery
DSMB: Data Safety Monitoring Board
HCW: Healthcare worker
LMIC: Low- and middle-income countries
IPV: Interpersonal violence
IRB: Institutional Review Board
KI: Key informant
MISAU: Mozambique Ministry of Health
NIH: National Institutes of Health
NICHD: National Institute of Child Health & Human Development
NPF: Never pregnant females
OHRP: Office for Human Research Protections
PID: Participant ID
PLHIV: People living with HIV
RCT: Randomized control trial
SAP: Statistical Analysis Plan
SOC: Standard of care
SRH: Sexual and reproductive health
SPIRIT: Standard Protocol Items Recommendations for Interventional Trials
SSA: Sub-Saharan Africa
US: United States
VL: Viral load
VS: Viral suppression
