# Correction to: The CombinADO study to assess the impact of a combination intervention strategy on viral suppression, antiretroviral therapy adherence, and retention in HIV care among adolescents and young people living with HIV: protocol for a cluster-randomized controlled trial

**DOI:** 10.1186/s13063-022-06008-2

**Published:** 2022-01-17

**Authors:** Phepo Mogoba, Maia Lesosky, Allison Zerbe, Joana Falcao, Claude Ann Mellins, Christopher Desmond, Carlos Arnaldo, Bill Kapogiannis, Landon Myer, Elaine J. Abrams

**Affiliations:** 1 Division of Epidemiology & Biostatistics, School of Public Health & Family Medicine, University of Cape Town, Level 5, Falmouth Building, Anzio Road, Cape Town, South Africa; 2 ICAP at Columbia University, Mailman School of Public Health, New York, USA; 3 HIV Center for Clinical and Behavioral Studies, Department of Psychiatry, New York State Psychiatric Institute and Columbia University Irving Medical Center, New York, NY USA; 4 Centre for Rural Health, University of KwaZulu Natal, Durban, South Africa; 5 Centro de Estudos Africanos, Universidade Eduardo Mondlane, Maputo, Mozambique; 6 Eunice Kennedy Shriver National Institute of Child Health and Human Development, Bethesda, MD USA; 7 Department of Pediatrics, Vagelos College of Physicians and Surgeons, Columbia University, New York, USA

**Correction to: Trials 22, 956 (2021)**

**https://doi.org/10.1186/s13063-021-05943-w**

Following the publication of the original article [1], we were notified of an error in the statistical analysis section. In line 555, in the sentence that begins with: “Planned subgroup analyses include by age group ( …)” , the age categories (i.e., 10–14, 15–19, and 20–24) incorrectly appeared as citations in the published article.

The original article has been corrected.
